# A Neuron Survival Protein May Give Directions, Too

**DOI:** 10.1371/journal.pbio.0020437

**Published:** 2004-11-23

Anyone who's ever had a physical exam knows the monosynaptic stretch reflex arc. When the doctor taps your patellar tendon with a hammer, your quadriceps muscle briefly stretches and your knee responds with a quick kick. This involuntarily reflex is mediated by muscle spindles, specialized muscle structures containing both proprioceptive (sensory) and motor neurons. Proprioceptive neurons send information about how much the muscle is stretched to the spinal cord, and motor neurons emanating from the spinal cord tell the muscle to contract, which corrects the stretch.

This reflex circuit is established in the developing embryo, when neurons migrate through and around developing tissues and send their axons to their signaling targets. Many neurons will successfully extend their axons into a target—which might be a muscle, another neuron, or some other tissue—but not all survive the process. Whether a neuron lives or dies depends on a family of growth factor proteins called neurotrophins. If a sensory neuron doesn't get enough neurotrophin-3 (NT-3), it will die.

Proper development of the sensory/motor circuit also depends on NT-3, which is expressed in limb buds, muscle spindles, and the ventral spinal cord: muscle spindle development depends on sensory axons, and motor neuron connections depend on developing limb buds. It has not been clear, however, whether NT-3 simply ensures the survival of proprioceptive neurons or whether it also helps establish the proprioceptive reflex arc. In a new study, Reha Erzurumlu and colleagues demonstrate a clear role for NT-3 in axon guidance.[Fig pbio-0020437-g001]

**Figure pbio-0020437-g001:**
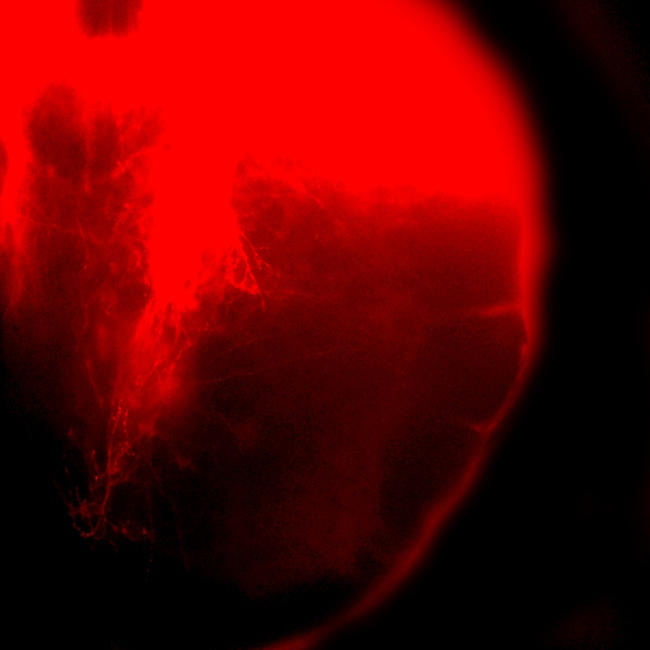
Developing nerves in the mouse spinal cord

Attempts to investigate the axon guidance theory have been difficult since sensory neurons die without NT-3. To circumvent this problem, the authors developed a “double knockout” mouse model that deletes both the NT-3 gene and the apoptosis-promoting gene *Bax*, which activates the cell death pathway for sensory neurons. This system removes NT-3 signaling without killing the sensory neurons, so the researchers can investigate any effects NT-3 may have on axon behavior.

Erzurumlu and colleagues showed that sensory neurons, in the absence of NT-3 signaling, project to the right places but never reach their final destination. In normal development, sensory neuron axons travel into the ventral spinal cord and form synapses with motor neuron dendrites in the ventral horn to establish the reflex arc circuit. In the double knockout mice, sensory neurons manage to extend into the spinal cord, but then get lost; they can't find the ventral horn, so they never form a synapse with the motor neuron dendrites. The failure to establish connections between the sensory axons and motor neurons in mice lacking NT-3, the authors argue, indicates that NT-3 is required for proper axon targeting.

Similarly, deprived of NT-3 signaling, sensory neuron axons fail to reach their ultimate targets in peripheral muscle. They project down toward the muscle but don't recognize the muscle and thus cannot enter or innervate it. Consequently, the muscles' sense organs, the muscle spindles, cannot differentiate. The authors argue that these findings, along with the results of tissue culture experiments, show that NT-3 acts as a short-distance cue for proprioceptive axons, which travel in the right direction but ultimately lose their way without NT-3.

Altogether these results show that proprioceptive axons require NT-3 not just for survival, but to reach their proper endpoints in the peripheral and central nervous system. NT-3 also helps proprioceptive axons initiate muscle innervation and spindle differentiation. Researchers developing therapies to treat neurodegenerative injuries have increasingly focused their attentions on growth factors like NT-3. By identifying the molecules and mechanisms that establish connections between sensory and motor neurons during development, it may be possible to engage similar processes to attenuate neurodegeneration and even repair damaged nerves.

